# Brachial artery injury following opened elbow dislocation associated with accessory brachial artery: two rare entities in a 17-year –old girl: case report

**DOI:** 10.11604/pamj.2015.20.265.3915

**Published:** 2015-03-19

**Authors:** Rita Hajji, Youssef Zrihni, Hamza Naouli, Abdellatif Bouarhroum

**Affiliations:** 1 Department of Vascular Surgery, UHC Hassan II Fes, Morocco

**Keywords:** Elbow, dislocation, arterial injury, accessory brachial artery

## Abstract

Elbow dislocations are the most frequently encountered after shoulder dislocations. In their vast majority, these injuries carry a good prognosis. Although, concomitant arterial injury is rare and make them more serious. In this paper, we report a case of a 17 year old woman with opened elbow dislocation with arterial injury associated to an artery variation: "accessory brachial artery"

## Introduction

Elbow dislocations are the most frequent after shoulder dislocations. Ninety percent of elbow dislocations are posterior or posterolateral [1]. The vascular lesions risk is probably related to the anatomic proximity of the periarticular neurovascular structures which makes concomitant lesions possible [2]. Adequate treatment of this injury includes reduction of dislocation, vascular repair and fixation of the elbow.

## Patient and observation

**Figure 1:**
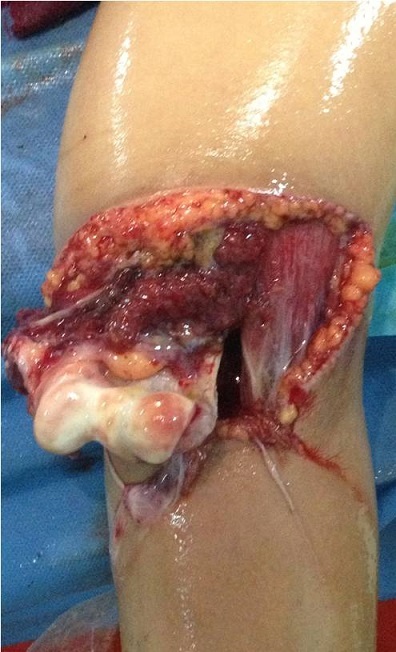
Opened elbow dislocation

**Figure 2:**
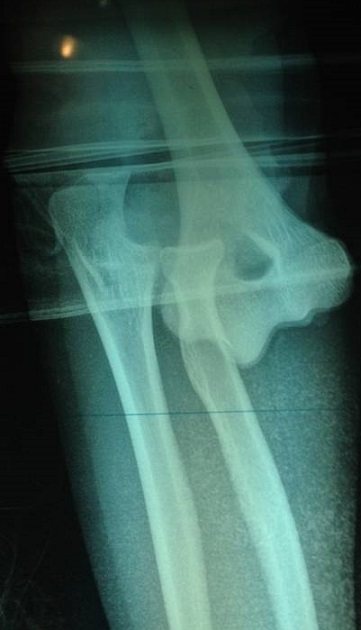
Radiograph of left posterior elbow dislocation

**Figure 3:**
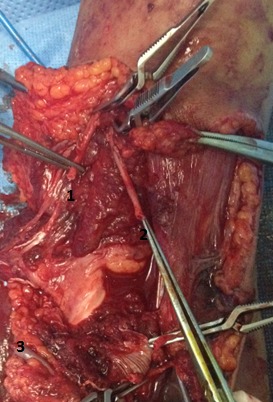
Intraoperative aspect of disruption of both brachial artery and accessory brachial artery

**Figure 4:**
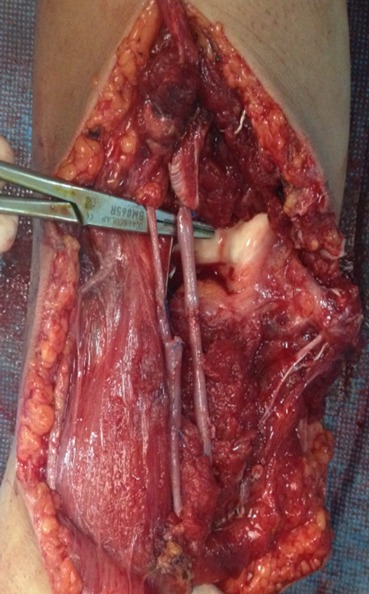
Final result of vein grafts

A 17 year-old girl with a dominant right hand fell from the third floor and landed on the left hand. She was admitted to our hospital one hour later. Opened elbow dislocation was noticed (Figure 1); No radial pulse was detectable. Radiographic examination revealed a posterior elbow dislocation (Figure 2). Arteriography was not performed because of the open injury required surgical exploration very soon. The patient underwent surgery, the dislocation was reduced. Exploration of the vascular axis showed a complete disruption of the brachial artery and the vein above and the basilic vein at the same level as the aponevrotic expansion of the biceps (Figure 3). In addition to this, exploration revealed another arterial structure. It seemed to be an accessory brachial artery because of its course on the medial side of the arm. The injury of both brachial artery and accessory brachial artery was complete. Two brachial antebrachial shunts were placed by using the ipsilateral basilic vein (Figure 4). The median nerve section was repaired. After vascular repair, instability after reduction was treated with an elbow cross pinning fixation by the traumatology team. After three weeks, pins were removed and passive mobilization of the elbow was begun.

## Discussion

The Frequency of arterial injury associated with dislocations remains difficult to establish because the literature reports short series of sporadic studies from traumatology teams. The patient groups are larger when they are reported by teams of vascular surgeons: Endean observed 8 arterial lesions out of 63 elbow dislocations [3]. Sparks cared for three artery lesions out of 634 elbow dislocations over 5 years [4]. The risk of vascular lesions is probably related to how far the displacement extends. In our case, vascular surgery is due to opened dislocation with high energy trauma. Most authors recommend arteriography in order to define the anatomical location of the lesions. In our case, dislocation was opened an the arterial injury seemed obvious [5, 6]. Concomitant involvement to the median or ulnar nerve is related to their vascular axis proximity. The arterial lesions observed are: complete or subadventitial rupture, incarceration and thrombosis [6] and vascular repair depends of this. In this reported case, the disruption was complete. Furthermore, we needed to cut into the healthy area to prevent risk of secondary thrombosis. So, vascular repair required a venous graft. The future tension on artery trunk during elbow extension must be planned also. We used the ipsilateral basilic vein instead a great saphenous graft because the basilic vein was injured. In our case, exploration showed also an arterial variation: an accessory brachial artery. This term was first established by Mc Cormack en 1953 [7]; It is rare upper limb vascular abnormality. The embryological origin of such variations in the vasculature of the upper limb may be explained as an abnormal deviation in the normal vascular patterns. The proximal part of the right subclavian artery (*arteria subclavia dextra*) arises from the right aortic arch and the distal part of the artery is derived from the right seventh intersegmental artery (*arteria intersegmentalis septima*). The left subclavian artery has a different embryological background, however, as the entire artery is formed from the seventh intersegmental artery [8]. The accessory brachial artery is derived from another embryological abnormality, referred to as the superficial brachial artery (*arteria brachialis superficialis, SBA*). The SBA is based on the persistence of more than one intersegmental cervical artery, which does not deteriorate but instead remains and can even grow larger, as stated by Jurjus [9]. So, we must know about brachial artery variations, especially during arm surgery, traumatic injuries, and trans radial/transulnar catheterization.

## Conclusion

Elbow dislocations are frequent and benign injuries. Their association with an arterial lesion is very rare, but should be systematically sought before and after reduction. So, at the least doubt, arteriography must be performed because of an incomplete thrombus may not be obvious because of the rich network around the elbow. Arteriography is required also to detect arterial variations. Management of these injuries must include reduction of the elbow, vascular repair an elbow fixation.
